# Neuromodulatory effects of theta burst stimulation to the prefrontal cortex

**DOI:** 10.1038/s41597-022-01820-6

**Published:** 2022-11-21

**Authors:** Adriano H. Moffa, Tjeerd W. Boonstra, Ashley Wang, Donel Martin, Colleen Loo, Stevan Nikolin

**Affiliations:** 1 School of Psychiatry, Black Dog Institute, University of New South Wales, Sydney, Australia. Hospital Rd, Randwick, Sydney, NSW 2031 Australia; 2 Faculty of Psychology and Neuroscience, Maastricht University, Universiteitssingel 40, 6229 ER Maastricht, Netherlands; 3 University of New South Wales, Sydney, Australia

**Keywords:** Neural circuits, Electroencephalography - EEG

## Abstract

Theta burst stimulation (TBS) is a new form of repetitive transcranial magnetic stimulation (TMS) capable of non-invasively modulating cortical excitability. In recent years TBS has been increasingly used as a neuroscientific investigative tool and therapeutic intervention for psychiatric disorders, in which the dorsolateral prefrontal cortex (DLPFC) is often the primary target. However, the neuromodulatory effects of TBS on prefrontal regions remain unclear. Here we share EEG and ECG recordings and structural MRI scans, including high-resolution DTI, from twenty-four healthy participants who received intermittent TBS (two sessions), continuous TBS (two sessions), and sham stimulation (one session) applied to the left DLPFC using a single-blinded crossover design. Each session includes eyes-open resting-state EEG and single-pulse TMS-EEG obtained before TBS and 2−, 15−, and 30-minutes post-stimulation. This dataset enables foundational basic science investigations into the neuromodulatory effects of TBS on the DLPFC.

## Abstract (summary)

**Table:** 

Measurement(s)	TMS-evoked potential • Diffusion Weighted Imaging • T1-weighted structural scan
Technology Type(s)	electroencephalography (EEG) • Magnetic Resonance Imaging • 3 T MRI scanner
Factor Type(s)	condition
Sample Characteristic - Organism	Homo sapiens
Sample Characteristic - Environment	Laboratory environment
Sample Characteristic - Location	Australia

## Background & Summary

Repetitive transcranial magnetic stimulation (rTMS) is a non-invasive form of brain stimulation that uses focal electromagnetic fields to elicit neuronal action potentials in the brain^1,2^. Depending on the frequency of these repetitive magnetic pulses, rTMS can induce local and downstream cortical neuromodulatory effects^3^. This capability has made rTMS an important neuroscientific investigative tool used to probe the cognitive and behavioural correlates of stimulated brain regions^4,5^. Additionally, rTMS delivered over multiple sessions can produce cumulative lasting changes in brain activity, which has shown immense promise as a therapeutic intervention for a range of neurological and neuropsychiatric conditions^2^.

Theta burst stimulation (TBS) is a relatively recent advance in rTMS that uses a pattern of magnetic pulses that mimic brain oscillatory activity. Specifically, rapid bursts of gamma activity (50 Hz) are enveloped within slow-wave theta (5 Hz) oscillations^6^. Intermittent theta-burst stimulation (iTBS) has been shown to increase motor cortical excitability, whereas continuous theta-burst stimulation (cTBS) produces cortical inhibitory effects^7,8^.The neuromodulatory properties of these forms of TBS are thought to be equivalent to, or larger than, rTMS^9,10^. Indeed, iTBS delivered to the prefrontal cortex produces non-inferior antidepressant effects compared to standard rTMS treatments^11^. A further benefit of TBS is that it can be delivered in a fraction of the time compared to standard rTMS (typically ~3 minutes), which presents significant advantages for research and clinical settings.

The mechanism by which TBS achieves such potent neuroplastic effects in such a short span of time remains unclear, particularly in the prefrontal cortex, a key region for therapeutic applications^11–16^. Furthermore, the literature has highlighted large inter- and intra-individual variability for effects of TBS in the motor cortex^17–19^. Electroencephalography (EEG) can potentially be used to assess the neuromodulatory mechanism of action of TBS and the underlying moderators of the observed heterogeneity.

Here we provide a rich EEG dataset containing both resting-state recordings and concurrent single-pulse TMS-EEG to explore changes in cortical activity induced by iTBS and cTBS applied to the left dorsolateral prefrontal cortex (DLPFC) in healthy subjects. This dataset was used in a study of the reliability of TBS and single-pulse TMS^20^, and a study of the neuromodulatory effects of TBS^21^. Resting-state data can be used to assess changes in functional connectivity metrics and determine whether TBS alters oscillatory activity within the frequency bands that comprise this type of intervention (i.e., 5 Hz and 50 Hz). TMS-EEG can be used to probe the local and downstream effects of TBS to distal functionally connected brain regions^22^. This dataset also contains repeated sessions of cTBS and iTBS, allowing for investigations of the test-retest reliability of TBS effects at the prefrontal cortex. Finally, the dataset incorporates individual structural magnetic resonance imaging (MRI) scans of the participants and includes neuronavigated coordinates of EEG electrodes to allow for precise source localisation and reconstruction analyses.

## Methods

### Participants

Twenty-four healthy adult participants (11 females, mean age 25.2 ± 9.9 years, ranging from 18 to 65) completed the study. Participants were compensated $50 AUD as reimbursement for time and expenses involved in attending each 2.5-hour study session, in addition to $10 AUD for the initial screening session, and $20 AUD for the MRI scan. All subjects were right-handed, as assessed by the Edinburgh Handedness Inventory^23^ and were excluded if they met any of the following criteria: (1) past psychiatric and neurological disorders including seizures and strokes; (2) recent head injury; (3) concurrent medication use affecting mental performance; (4) drug or alcohol abuse in the last three months; (5) smokers; (6) currently pregnant; and (7) had any contraindications for EEG and MRI. None of the participants were taking contraceptives at the time of screening and enrolment in the study. The phase of the menstrual cycle was not assessed in this experiment, although recent evidence has shown that this may influence TMS-EEG outcomes^24^. Written informed consent was obtained from all participants prior to starting the study, which was approved by the University of New South Wales Human Research Ethics Committee (HC17765).

### Experiment design

**Fig. 1:**
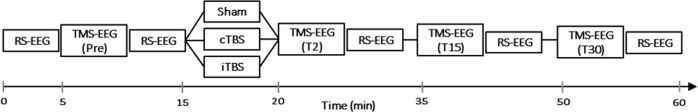
Experiment timeline. Blocks consisting of four minutes of eyes-open resting-state electroencephalography (RS-EEG) and 100 single pulses of transcranial magnetic stimulation (TMS-EEG) were acquired before and after the intervention consisting of either sham, continuous theta-burst stimulation (cTBS), or intermittent theta-burst stimulation (iTBS).

The study utilised a single-blinded sham-controlled crossover design. Participants underwent five sessions each at approximately the same time of the day with at least one week between sessions to avoid carryover effects. The order of sessions was pseudo-randomized using a computer-generated list. The first three sessions included a session each of iTBS, cTBS, and sham (in randomised order), followed by an additional session each of iTBS and cTBS (in randomised order). TMS-EEG was assessed in blocks of 100 single TMS pulses acquired pre-TBS and at 2-, 15-, and 30-min post-TBS (Pre, T2, T15 and T30, respectively). Eyes-open resting-state EEG data was recorded for four minutes at the start of the session and after all four blocks of single-pulse TMS as outlined in Fig. 1.

### Electroencephalography

EEG data were acquired using a Refa 2048 Hz EEG system (TMSi, Oldenzaal, the Netherlands) and an appropriately sized 64-channel 10–20 EEG cap (EasyCap, GmbH, Hernschig, Germany) as determined by head circumference, with sintered, interrupted disk, Ag-AgCl TMS-compatible electrodes. The position of the EEG cap was confirmed by matching the Cz electrode with the intersection of the participants’ nasion-inion and tragus-tragus axes. Electrodes were grounded to Fpz, and EEG signals were measured against a common average reference. To reduce scalp impedance, participants were instructed to wash their hair before attending each experiment session. Secondly, prior to cap placement, the participant’s scalp was cleaned with alcohol swabs. Lastly, an electro-conductive gel and blunted needles were used to lightly abrade the scalp to limit impedances to less than 50 kΩ, which is well below 1% of the input impedance (100MΩ) of the EEG amplifier^25^. Electrooculography (EOG) channels were placed superior and inferior to the right eye to capture vertical EOG (VEOG), and on the edge of the lateral canthi to capture horizontal EOG (HEOG). Electrocardiogram (ECG) electrodes were positioned below the right clavicle and on the left midclavicular line approximately at the 8–10^th^ false ribs. The 3-dimensional coordinates of scalp electrode positions were acquired using neuronavigational software, Xensor™ (ANT-Neuro, Hengelo, the Netherlands).

### Transcranial magnetic stimulation

TBS and single-pulse TMS were delivered using a MagPro® X100 (MagVenture Company, Lucernemarken, Denmark) with a 65 mm diameter Cool-B65 figure-8 stimulation coil. The coil was positioned tangentially to the scalp over the F3 electrode in order to target the DLPFC. A 5-mm customised 3D-printed spacer was placed between the coil and the scalp at all times to avoid contact with electrodes to minimise post-pulse artefacts, electrode movement, and bone-conducted auditory input. The coil was oriented at a 45-degree angle relative to the parasagittal plane, and the TMS pulse was delivered using a biphasic waveform. A hard foam headrest connected to a mechanical arm was positioned on the contralateral temporal region of the stimulation site to ensure minimal participant movement.

### Resting motor threshold

Resting motor thresholds (RMTs) were determined for each individual by identifying the lowest stimulus intensity required to elicit at least 3 out of 6 motor-evoked potentials with a peak-to-peak amplitude of at least 50 µV across the contralateral right first dorsal interosseous muscle as measured by EMG using a 1401 laboratory interface with a 1902 amplifier (Cambridge Electronic Design, Cambridge, UK) and the Signal V4 data acquisition package (Cambridge Electronic Design, Cambridge, UK). RMT was assessed at the first visit only and was not re-evaluated prior to each stimulation session. The mean RMT for participants was 65.9% (SD = 6.7) of maximal stimulator output (MSO) with the spacer over the EEG cap, and 47.3% (SD = 5.1) with the TMS coil placed directly on the participant’s scalp. The stimulation intensity was titrated to 120% of each subject’s RMT for single-pulse TMS and 75% RMT for TBS.

### Single pulse transcranial magnetic stimulation

TMS-EEG acquisition involved 100 pulses delivered once every 4 seconds with a 10% jitter. Pulses were triggered using a custom-written MATLAB script via a falling-edge protocol using a National Instruments Card (NIUSB6259, National Instruments, Austin, USA). The trigger pulses controlling the TMS device were recorded together with the EEG to enable event-related averaging.

### Theta burst stimulation

The iTBS protocol utilised two-second trains of TMS delivered every 10 seconds for a total of 192 seconds (600 pulses), while the cTBS protocol delivered 600 pulses continuously over 40 seconds^7^. The sham condition utilised an inactive coil placed on the head. A second coil was positioned 20 cm posterior facing away from the subject to provide auditory stimulation and thereby improve blinding. The stimulation intensity of this posterior coil was increased by 20% to compensate for the increased distance away from the ear. The posterior coil simulated an iTBS protocol in half the subjects and a cTBS protocol in the other half. To further improve blinding, subjects were provided white noise through earphones at a volume sufficient to mask the auditory stimulation of the TMS clicking sound or at the limit of the participant’s comfort. In addition, earmuffs were placed over the earphones to further attenuate TMS pulse volume during the TMS-EEG blocks.

### Tolerability

Overall, iTBS and cTBS interventions were well tolerated, with the most common side effect being a moderate headache. Adverse events during iTBS included moderate headache (n = 2) and mild nausea (n = 1). During cTBS participants reported mild blurred vision (n = 1), moderate headache and fatigue (n = 1), mild nausea and moderate dizziness (n = 1). During sham stimulation one participant reported moderate fatigue (n = 1). No participants dropped out of the study.

### Resting state

Each resting-state block lasted for four minutes. During resting-state recordings, participants were instructed to stay relaxed, keep their eyes open, and look at a fixation cross presented in the middle of a 58.4 cm (23-inch) computer screen in a dimly lit, sound-attenuated room.

### Structural MRI scan acquisition

The MRI scans were obtained using a Philips Achieva 3 T (TX) - DS MRI scanner based at Neuroscience Research Australia (NeuRA), Sydney, Australia. A single scan was obtained for each participant at baseline prior to the start of the TMS sessions. All MRI scans have been de-identified and anonymised using the Fieldtrip *ft_defacevolume* and *ft_anonymizedata* functions. For all participants, T1-weighted sequences (TR = 5.7 ms, TE = 2.6 ms, FOV = 250 × 250 × 190 mm, voxel size = 1 × 1 × 1 mm, matrix 250 × 250, Flip angle 8°, 190 sagittal plane slices) were used to acquire structural MR images covering the whole brain. In the same session, high-resolution DTI (TR = 13737 ms, TE = 59 ms, FOV = 240 × 240 × 120 mm, voxel size = 2 × 2 × 2 mm, matrix 120 × 120, Flip angle 90°, 30 transverse plane slices) was also acquired. Researchers may use the present dataset to assess how individual differences in brain morphology, including white matter fibre bundle size, grey matter volume, and whole brain volume, may affect the TMS-evoked potential.

### EEG acquisition and pre-processing

Data were cleaned and analysed offline, blind to the experimental condition, using a combination of open-source toolboxes: Fieldtrip^26^, EEGLAB^27^, TESA (v0.1.0)^28^ and custom scripts on the MATLAB platform (R2017b, The MathWorks, USA).

### TMS-evoked potentials

**Fig. 2:**
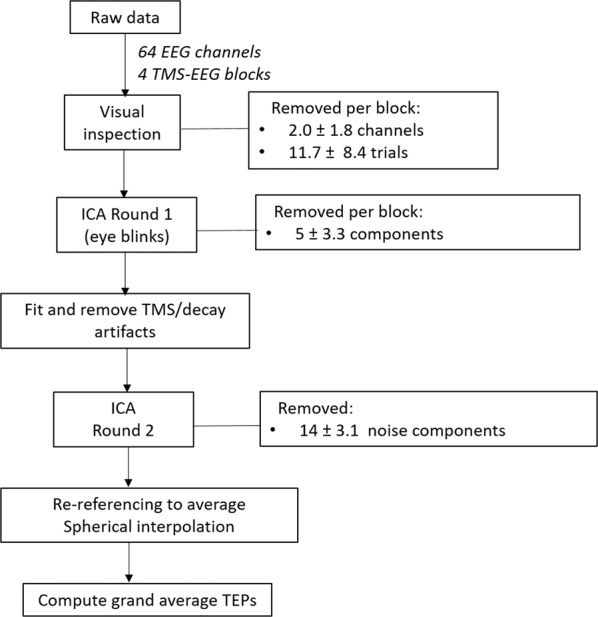
Data processing flowchart. TMS-EEG: transcranial magnetic stimulation-electroencephalography; ICA: independent component analysis; TMS: transcranial magnetic stimulation; TEPs: TMS-evoked potentials.

The cleaning parameters and procedures were based on the method described in Rogasch, *et al*.^28^. Data were epoched around the TMS pulse (−1000 to 1000 ms). Electrodes in which the TMS artefact exceeded the maximum absolute value of the range of the amplifier (10^7^ μV) were removed and linearly interpolated from neighbouring channels. An average of 2.0 ± 1.7 channels was rejected in the cTBS condition, 2.1 ± 1.8 channels in the iTBS condition and 2.0 ± 1.8 channels in the sham condition. Trials were excluded if kurtosis exceeded five standard deviations from the mean. The remaining trials were visually inspected, and trials with excessive noise (e.g., muscle activity, electrode artefacts) were discarded (Fig. 2). An average of 11.2 ± 12.5 trials were rejected per block in the cTBS condition, 12.5 ± 6.1 trials in the iTBS condition and 11.5 ± 6.6 trials in the sham condition (out of 100 trials).

EEG traces were detrended and baseline-corrected relative to pre-TMS data (−500 to −50 ms). Line noise (50 Hz) was removed using linear regression by fitting and subtracting a sine wave from the EEG. Data between −5 and 10 ms around the TMS pulse were removed, and an initial round of independent component analysis (ICA) was performed using the TESA *compselect* function to eliminate components containing eye blinks. On average, 5.0 ± 3.3 components were removed at this stage. Next, TMS-muscle and decay artefacts were removed using a method proposed by Freche, *et al*.^29^, in which a physical model of skin impedance is used to remove the artefactual signal without degrading the neural signal. This was achieved by fitting a power law to the most negative and positive EEG signal deflections caused by the TMS artefact to obtain regression fit parameters, and then removing the artefact from the data by subtraction^29^.

Following removal of the TMS-muscle and decay artefacts, EEG data from before and after the TMS stimulus were filtered separately using a bandpass filter (1–90 Hz). A more lateral stimulation site such as F3 can induce muscle activation and electrode movements^30,31^, and a second round of ICA was performed to remove this as well as components associated with blinks, eye movement, persistent muscle activity, decay artefacts and electrode noise. A total of 14.0 ± 3.1 components were removed at this stage. Finally, the EEG was re-referenced to the common average reference and trials were separately averaged within each experiment block to obtain the TEPs.

### Resting state

Eyes-open resting-state EEG data were down-sampled to 512 Hz, baseline-corrected (demeaned) and detrended (i.e., any linear trend was removed). The data was then filtered using a second-order bandpass filter (0.1–70 Hz) and a notch filter at 50 Hz to remove electrical line noise. Data were epoched in 1-s intervals. Epochs were firstly rejected using an automated algorithm in which epochs with data ranges greater than three standard deviations or absolute maxima greater than 12 standard deviations of other epochs were removed. A visual inspection was used to reject any remaining noisy epochs. A single round of ICA was used to remove components containing eye blinks and muscle artefacts. Following ICA, EEG data were re-referenced to the common average reference. Power spectral densities (PSD) were calculated using 180 seconds of data (the largest time window shared by all blocks after bad epochs were removed). Log-normalised power spectral density values (μV^2^/Hz) were estimated for each EEG electrode over a range of 1–70 Hz using the fast Fourier transform with 2-second sliding Hamming windows with 50% overlap, as initially described by Welch^32^.

## Data Records

The dataset consists of 119 separate EEG recording sessions (1 session was excluded due to technical issues). All data were de-identified, and participants provided written informed consent for their anonymised de-identified data to be shared publicly. The raw data files and code can be accessed via the FigShare open access repository service (10.25452/figshare.plus.c.5910329)^33^, which have been made available under the Attribution 4.0 International Creative Commons License.

The dataset is stored and labelled as per the Brain Imaging Data Structure^34^. Subject files are named according to the following format:

$${\prime\prime} {\rm{sub}} \mbox{-} {\boldsymbol{A}}{\boldsymbol{A}}\_{\rm{ses}} \mbox{-} {\boldsymbol{B}}{\boldsymbol{B}}\_{\rm{task}} \mbox{-} {\boldsymbol{B}}{\boldsymbol{L}}{\boldsymbol{O}}{\boldsymbol{C}}{\boldsymbol{K}}{\boldsymbol{T}}{\boldsymbol{I}}{\boldsymbol{M}}{\boldsymbol{E}} \mbox{-} {\boldsymbol{B}}{\boldsymbol{L}}{\boldsymbol{O}}{\boldsymbol{C}}{\boldsymbol{K}}{\boldsymbol{T}}{\boldsymbol{Y}}{\boldsymbol{P}}{\boldsymbol{E}}\_{\rm{run}} \mbox{-} {\boldsymbol{C}}{\boldsymbol{C}}.{\rm{ext}},$$

where ***AA*** is the subject number (01, 02, …, 24), ***BB*** is the session number (01, 02, …, 05), ***BLOCKTIME*** refers to whether the block occurred before/after the TBS intervention, ***BLOCKTYPE*** is the task (resting-state or TMS-EEG), ***CC*** is the order of the task presentation (01, 02, 03), and*.ext* is the file format (.csv,.mat,.nii, etc…).

EEG data files are stored in.mat format and contain raw EEG data, which can be accessed using MATLAB-toolboxes such as Fieldtrip^26^, EEGLAB^27^ or TESA (v0.1.0)^28^. The data in each file includes 68 labelled signals (64 EEG channels, ECG, HEOG, VEOG, and a Trigger channel to mark events). Experiment blocks are labelled using the naming structure shown in Table 1. Additional files include a generic description of the metadata (dataset_description.csv), participant demographic and stimulation parameter details (participants.csv), metadata of the experiment tasks and EEG recording system (eeg.csv), a list of all EEG channels (channels.csv) and their neuronavigated coordinates (electrodes.csv). Lastly, source data is provided in the MATLAB file format (.mat).

**Table 1:** Naming structure of MRI scans and EEG task blocks for each session.

Experiment Block Sequence	Filename Format
3D Ultrashort Echo Time sequence	3DUTESkull2mmiso
Susceptibility weighted imaging	sWIP3DUTESkull2mmiso
Diffusion Tensor Imaging	DTI
T1-weighted image	T1075TFESag
Eyes-open resting-state EEG	pre-rest_run-01
Single-pulse TMS-EEG	pre-tep_run-01
Eyes-open resting-state EEG	pre-rest_run-02
cTBS/iTBS/Sham	tbs
Single-pulse TMS-EEG	post-tep_run-01
Eyes-open resting-state EEG	post-rest_run-01
Single-pulse TMS-EEG	post-tep_run-02
Eyes-open resting-state EEG	post-rest_run-02
Single-pulse TMS-EEG	post-tep_run-03
Eyes-open resting-state EEG	post-rest_run-03

## Technical Validation

**Fig. 3:**
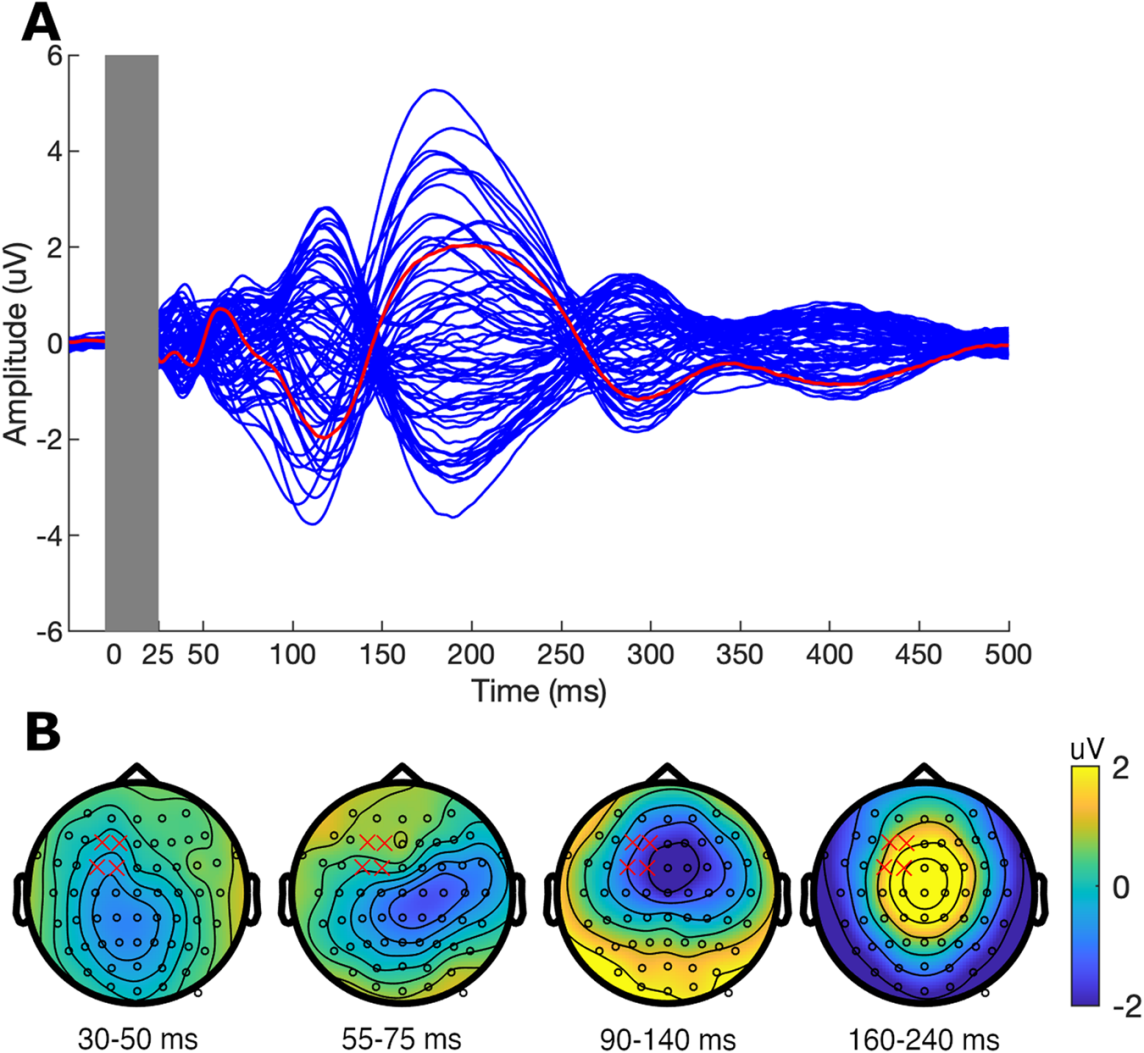
Transcranial magnetic stimulation evoked potentials (TEPs). Single-pulse TMS over the left dorsolateral prefrontal cortex (L-DLPFC) at baseline (i.e., pre-TBS). TEPs were combined across the five sessions and averaged across all participants. (**A**) Butterfly plot from all electrodes. The red line indicates the waveform obtained from the mean of four electrodes (F3, FC3, F1, FC1) near the stimulation site. The grey box indicates removed data points due to TMS-related artefacts and cleaning steps. (**B**) Topographical distribution for each peak of interest averaged across the time indicated below. Red crosses indicate the four electrodes comprising the region of interest (F3, FC3, F1, FC1).

**Fig. 4:**
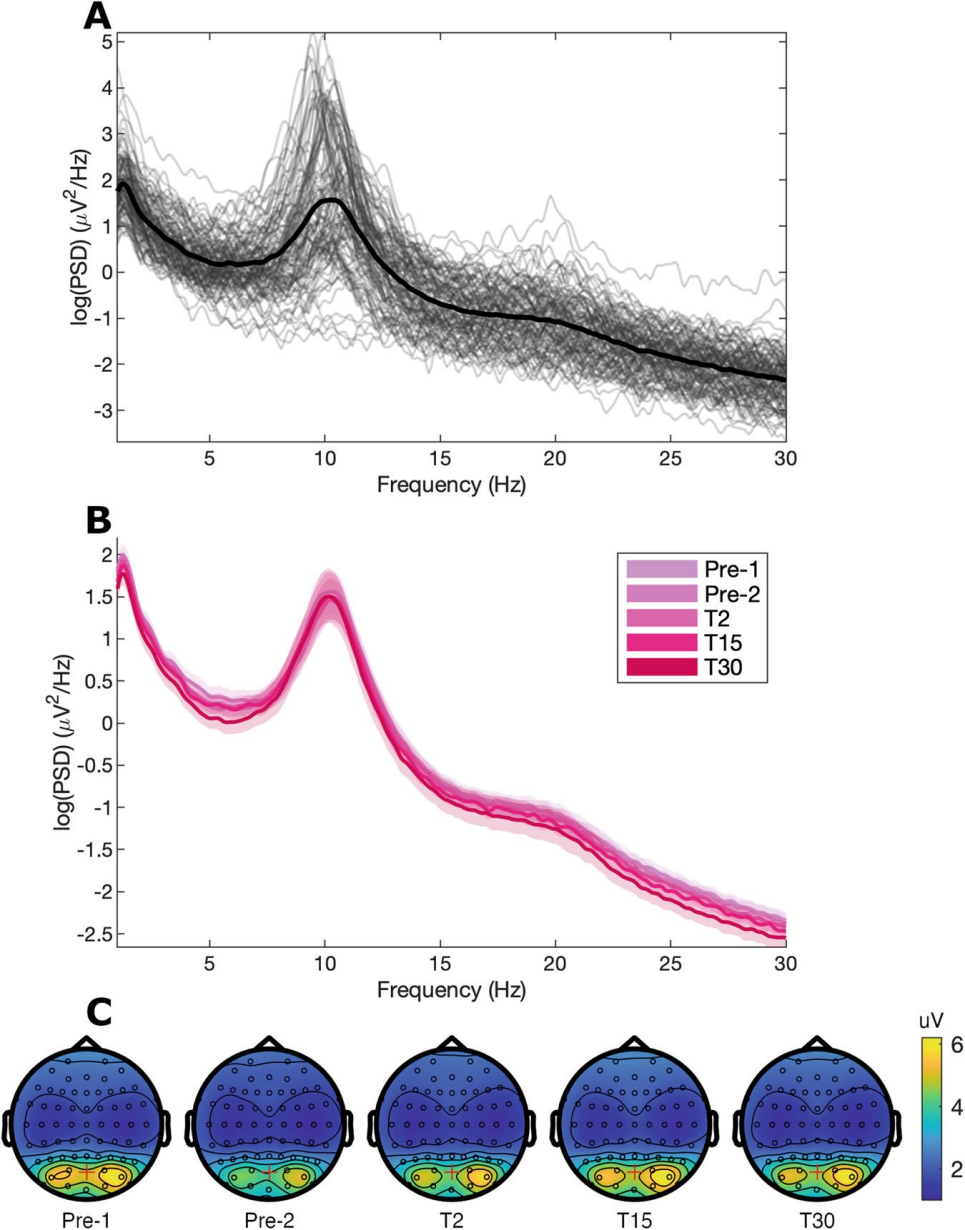
Eyes-open resting-state power spectral density. (**A**) Individual power spectral density (PSD) plots of all participants at baseline (Pre-1) at channel POz. (**B**) Power spectra for all time points with bootstrapped 95% confidence intervals at channel POz, demonstrating good agreement in power spectra obtained from different time points. (**C**) Topography for alpha (8–12 Hz) power. The red cross indicates channel POz.

We provide the grand average of all subjects across all baseline sessions for TEP waveforms (Fig. 3) and eyes-open resting-state (Fig. 4).

### TMS evoked potentials

The grand average of the TEP waveforms at the pre-TBS block is illustrated in Fig. 3A. Single-pulse TMS over the left DLPFC generated a series of deflections in the EEG traces, including two negative (N40, N100) and two positive (P60 and P200) components, classified according to their latencies after the TMS pulse^35–37^. Each component showed a distinctive topology (Fig. 3B), consistent with previous TMS-EEG studies in the prefrontal cortex^35,37,38^.

### Resting state

Eyes-open resting data show a clear alpha peak at approximately 10.3 Hz (Fig. 4A), which is consistent across the experiment blocks (Fig. 4B).

## Usage Notes

All files were sampled at 2048 Hz except for session 3 from subject 9 (sham condition), which was sampled at 1024 Hz due to technical reasons. Three sessions were recorded in two separate files due to technical issues (session 5 from subject 8; session 1 from subject 12 and session 2 from subject 17). These are provided as a single concatenated file in the source data subfolder, and relevant experiment blocks are provided similar to other session data. Session 1 from subject 14, and session 5 from subject 21, presented technical problems in all resting-state blocks; Session 5 from subject 11 and session 1 from subject 13 presented technical problems in all resting-state blocks except baseline and were excluded from the resting-state analyses; Session 1 from subject 12 does not have the last resting-state block due to technical issues.

Despite use of a spacer separating the TMS coil from EEG recording electrodes to avoid contact, channels immediately beneath the TMS coil (i.e., F3 and surrounding channels) tended to present increased noise. This is likely due to stimulation artefacts associated with single-pulse TMS as well as contact pressure between the coil and the EEG cap introducing movement artefacts. Sessions in which the F3 electrode was excluded (and interpolated by surrounding electrodes) due to artifact or excessive noise include: session 4 of subject 5, session 2 of subject 10, sessions 2 and 4 of subject 16, session 5 of subject 17, and lastly sessions 2, 3 and 5 of subject 21.

The following is a qualitative description of sessions in which we identified significant artefactual or noisy EEG recordings. For single-pulse TMS-EEG, sessions with more than 20% of rejected trials in at least one TMS-EEG block include: session 3 from subject 1, session 5 from subject 10, session 5 from subject 11, session 1 from subject 14, session 3 from subject 19 and session 4 from subject 23. For eyes-open resting-state EEG, sessions with less than 180 s of resting-state data remaining in at least one block following exclusion of rejected trials include: session 3 from subject 1, sessions 3, 4 and 5 from subject 9, session 5 from subject 10, session 1 from subject 11, sessions 2, 3 and 5 from subject 14, session 2 from subject 12, session 3 from subject 16, session 3 from subject 17, sessions 2, 3 and 5 from subject 18 and session 4 from subject 21.

## Data Availability

All custom-written MATLAB scripts used for EEG processing and calculation of neurophysiological measures are included in the ‘code’ folder of the provided dataset.

## References

[1] Pascual-Leone, A. *et al*. Safety of rapid-rate transcranial magnetic stimulation in normal volunteers. **89**, 120–130 (1993). 10.1016/0168-5597(93)90094-6 7683602

[2] Pascual-Leone A, Valls-Solé J, Wassermann EM, Hallett M (1994). Responses to rapid-rate transcranial magnetic stimulation of the human motor cortex. Brain.

[3] Wassermann E (1997). Local and distant changes in cerebral glucose metabolism during repetitive transcranial magnetic stimulation (rTMS). Neurology.

[4] Paus T (2005). Inferring causality in brain images: a perturbation approach. Philosophical transactions of the Royal Society of London. Series B, Biological sciences.

[5] Sack AT (2006). Transcranial magnetic stimulation, causal structure–function mapping and networks of functional relevance. Current opinion in neurobiology.

[6] Huang Y-Z, Rothwell JC (2004). The effect of short-duration bursts of high-frequency, low-intensity transcranial magnetic stimulation on the human motor cortex. Clinical neurophysiology.

[7] Huang Y-Z, Edwards MJ, Rounis E, Bhatia KP, Rothwell JC (2005). Theta burst stimulation of the human motor cortex. Neuron.

[8] Wischnewski M, Schutter DJ (2015). Efficacy and time course of theta burst stimulation in healthy humans. Brain Stimul.

[9] Nyffeler T (2006). Repetitive TMS over the human oculomotor cortex: comparison of 1-Hz and theta burst stimulation. Neuroscience letters.

[10] Yang W (2015). Comparison of different stimulation parameters of repetitive transcranial magnetic stimulation for unilateral spatial neglect in stroke patients. Journal of the Neurological Sciences.

[11] Blumberger DM (2018). Effectiveness of theta burst versus high-frequency repetitive transcranial magnetic stimulation in patients with depression (THREE-D): a randomised non-inferiority trial. The Lancet.

[12] Grossheinrich N (2009). Theta burst stimulation of the prefrontal cortex: safety and impact on cognition, mood, and resting electroencephalogram. Biological psychiatry.

[13] Cho SS (2010). Continuous theta burst stimulation of right dorsolateral prefrontal cortex induces changes in impulsivity level. Brain Stimul.

[14] Lowe CJ, Manocchio F, Safati AB, Hall PA (2018). The effects of theta burst stimulation (TBS) targeting the prefrontal cortex on executive functioning: a systematic review and meta-analysis. Neuropsychologia.

[15] Ngetich R, Zhou J, Zhang J, Jin Z, Li L (2020). Assessing the effects of continuous theta burst stimulation over the dorsolateral prefrontal cortex on human cognition: a systematic review. Frontiers in integrative neuroscience.

[16] Fitzgerald PB, Chen L, Richardson K, Daskalakis ZJ, Hoy KE (2020). A pilot investigation of an intensive theta burst stimulation protocol for patients with treatment resistant depression. Brain Stimul.

[17] Hinder MR (2014). Inter-and intra-individual variability following intermittent theta burst stimulation: implications for rehabilitation and recovery. Brain Stimul.

[18] Vernet M (2014). Reproducibility of the effects of theta burst stimulation on motor cortical plasticity in healthy participants. Clinical Neurophysiology.

[19] Ozdemir RA (2021). Reproducibility of cortical response modulation induced by intermittent and continuous theta-burst stimulation of the human motor cortex. Brain Stimulation.

[20] Moffa AH, Nikolin S, Martin D, Loo C, Boonstra TW (2022). Reliability of transcranial magnetic stimulation evoked potentials to detect the effects of theta-burst stimulation of the prefrontal cortex. Neuroimage: Reports.

[21] Moffa, A. H., Nikolin, S., Martin, D., Loo, C. & Boonstra, T. W. Assessing neuromodulation effects of theta burst stimulation to the prefrontal cortex using TMS-evoked potentials. *bioRxiv* (2021). 10.1111/ejn.70121 PMC12044518 40308179

[22] Siebner HR (2009). Consensus paper: combining transcranial stimulation with neuroimaging. Brain Stimul.

[23] Oldfield RC (1971). The assessment and analysis of handedness: the Edinburgh inventory. Neuropsychologia.

[24] Chung SW (2019). The influence of endogenous estrogen on high-frequency prefrontal transcranial magnetic stimulation. Brain Stimul.

[25] Metting van Rijn A, Peper A, Grimbergen C (1991). High-quality recording of bioelectric events. Medical and Biological Engineering and Computing.

[26] Oostenveld R, Fries P, Maris E, Schoffelen J-M (2011). FieldTrip: open source software for advanced analysis of MEG, EEG, and invasive electrophysiological data. Comput Intell Neurosci.

[27] Delorme A, Makeig S (2004). EEGLAB: an open source toolbox for analysis of single-trial EEG dynamics including independent component analysis. Journal of neuroscience methods.

[28] Rogasch NC (2017). Analysing concurrent transcranial magnetic stimulation and electroencephalographic data: a review and introduction to the open-source TESA software. Neuroimage.

[29] Freche D, Naim-Feil J, Peled A, Levit-Binnun N, Moses E (2018). A quantitative physical model of the TMS-induced discharge artifacts in EEG. PLoS Comput Biol.

[30] Ilmoniemi RJ, Kičić D (2010). Methodology for combined TMS and EEG. Brain topography.

[31] Huang G, Mouraux A (2015). MEP latencies predict the neuromodulatory effect of cTBS delivered to the ipsilateral and contralateral sensorimotor cortex. PLoS One.

[32] Welch P (1967). The use of fast Fourier transform for the estimation of power spectra: a method based on time averaging over short, modified periodograms. IEEE Transactions on audio and electroacoustics.

[33] Nikolin S, Boonstra T, Moffa A (2022). Figshare.

[34] Pernet CR (2019). EEG-BIDS, an extension to the brain imaging data structure for electroencephalography. Scientific data.

[35] Conde V (2019). The non-transcranial TMS-evoked potential is an inherent source of ambiguity in TMS-EEG studies. Neuroimage.

[36] Chung SW, Rogasch NC, Hoy KE, Fitzgerald PB (2018). The effect of single and repeated prefrontal intermittent theta burst stimulation on cortical reactivity and working memory. Brain Stimul.

[37] Chung SW (2017). Demonstration of short-term plasticity in the dorsolateral prefrontal cortex with theta burst stimulation: A TMS-EEG study. Clinical Neurophysiology.

[38] Hill A, Rogasch N, Fitzgerald P, Hoy K (2017). Exploring the the neurophysiological effects of transcranial direct current stimulation over non-motor brain regions via EEG and TMS-EEG. Brain Stimulation: Basic, Translational, and Clinical Research in Neuromodulation.

